# Supplementary figures and images for: Afraid of the dark: Light acutely suppresses activity in the human amygdala
Source: PLoS One. 2021 Jun 16;16(6):e0252350. doi: 10.1371/journal.pone.0252350 (PMC8208532; doi:10.1371/journal.pone.0252350)

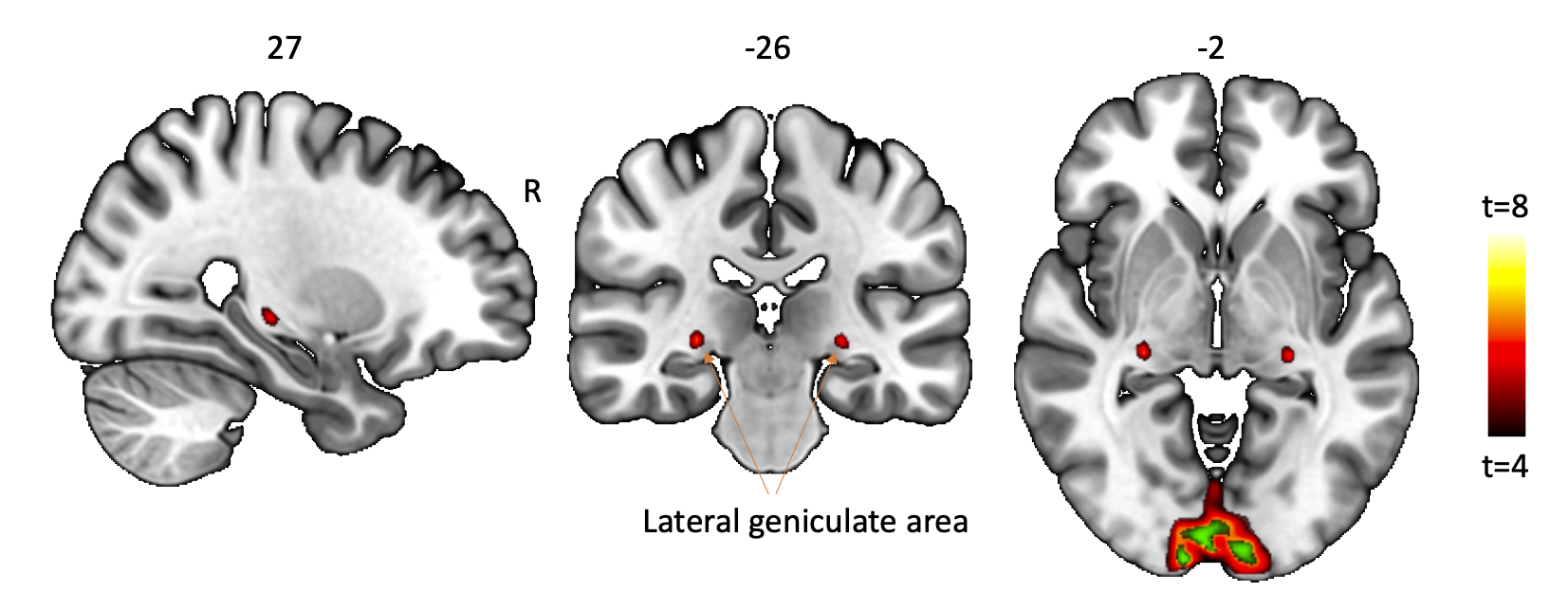

Supplement: S1 Fig — Increased activity (p<0.05, voxel-wise corrected) during light compared to dark was observed in the visual cortex (local maxima MNI coordinate: 14, -90, 2, tmax = 8.98) and right lateral geniculate area (tmax = 6.3, MNI coordinate: 26, -25, -2). Bilateral lateral geniculate area showed activation at p<0.001. However, this did not survive whole-brain correction for significance. Voxels with increased activity in the visual cortex and lateral geniculate area of the thalamus are shown. Green voxels represent the voxels significant at p<0.05 (voxel-wise corrected). Red-yellow color represent increased activity at p<0.001, uncorrected. The brain images are presented on a radiological orientation. (TIFF) [file pone.0252350.s001.tiff]
